# Ecology of Fungus Gnats (*Bradysia* spp.) in Greenhouse Production Systems Associated with Disease-Interactions and Alternative Management Strategies

**DOI:** 10.3390/insects6020325

**Published:** 2015-04-09

**Authors:** Raymond A. Cloyd

**Affiliations:** Department of Entomology, Kansas State University, Manhattan, KS 66506, USA; E-Mail: rcloyd@ksu.edu; Tel.: +1-785-532-4750; Fax: +1-785-532-6232

**Keywords:** fungus gnats, pest management, plant protection, diseases, greenhouse, cultural control, sanitation, physical barriers, repellents

## Abstract

Fungus gnats (*Bradysia* spp.) are major insect pests of greenhouse-grown horticultural crops mainly due to the direct feeding damage caused by the larvae, and the ability of larvae to transmit certain soil-borne plant pathogens. Currently, insecticides and biological control agents are being used successively to deal with fungus gnat populations in greenhouse production systems. However, these strategies may only be effective as long as greenhouse producers also implement alternative management strategies such as cultural, physical, and sanitation. This includes elimination of algae, and plant and growing medium debris; placing physical barriers onto the growing medium surface; and using materials that repel fungus gnat adults. This article describes the disease-interactions associated with fungus gnats and foliar and soil-borne diseases, and the alternative management strategies that should be considered by greenhouse producers in order to alleviate problems with fungus gnats in greenhouse production systems.

## 1. Introduction

Fungus gnats in the genus *Bradysia* spp. (Diptera: Sciaridae) are major insect pests of greenhouse production systems feeding on a wide-range of horticultural crops [1,2,3] with the commonly encountered species being *Bradysia coprophila* Comstock and *B. impatiens* Johannsen [4,5,6]. Fungus gnats, in general, are ubiquitous in greenhouses [7] but are primarily a problem under conditions of excessive moisture, which commonly occurs during propagation when cuttings and plugs are developing root systems [8,9]. The larval stage, which resides within the growing medium, feeds mainly on decaying plant material and fungi [10]. However, they will also feed on healthy plant roots and tunnel into stems of young cuttings and seedlings [4], and the crowns of mature plants [11]. Therefore, the larvae are primarily responsible for causing direct plant damage by feeding on the roots, thus interfering with the ability of plants to uptake water and nutrients, which results in wilting and stunted growth [3,4,12,13]. Fungus gnat adults are mainly a nuisance causing minimal direct plant damage [9].

A number of insecticides from different chemical classes and various biological control agents including a soil-dwelling predatory mite (*Stratiolaelaps scimitus* (Womersley)), a rove beetle (*Dalotia coriaria* Kraatz), and entomopathogenic nematode (*Steinernema feltiae* Filipjev) have been shown to be effective in suppressing or regulating fungus gnat populations [1,8,14,15,16,17,18,19]. However, these strategies may only be effective if alternative plant protection strategies are implemented simultaneously. Furthermore, sole reliance on insecticides may be an issue as there is the possibility of fungus gnats developing resistance to insecticides [20,21]. This article will discuss the disease-interactions associated with fungus gnats, and the alternative management strategies that should be considered so as to alleviate problems with fungus gnats in greenhouse production systems.

## 2. Disease-Interactions

Fungus gnat larvae not only cause direct damage during feeding but their feeding may predispose plants to attack from soil-borne plant pathogens by creating wounds, which allow entry of certain soil-borne fungi [12,22,23,24]. Furthermore, larvae are capable of directly transmitting certain fungal diseases including *Pythium* spp., *Fusarium* spp., and *Verticillium* spp., from diseased to non-infected plants [24,25,26,27]. It has been shown that fungus gnat larvae can transmit *Thielaviopsis basicola* Berk & Broome, *Fusarium oxysporum* f. sp. *radices-lycopersici* Jarvis & Shoemaker, *Verticillium albo-atrum* Reinke & Berthold, and *Fusarium avenaceum* (Fr:Fr) [24,27,28,29,30]. It has also been reported that the oospores of certain *Pythium* spp. may survive passage through the digestive tract of *B. impatiens* larvae, which might be intact/viable after being excreted [24]. Furthermore, larvae of the fungus gnat, *B. impatiens* have been shown to ingest the propagules of *Pythium aphanidermatum* (Edson) and macroconidia of *F. avenaceum* that can then be introduced into young healthy plants during the feeding process [13,30]. Under laboratory conditions, fungus gnat larvae are capable of transmitting *Pythium* spp.; however, the extent of larval transmission may be less of a factor under greenhouse conditions, and may vary depending on the specific *Pythium* species [31]. In addition, it has been suggested, based on laboratory experiments, that fungus gnat larval feeding may decrease geranium seedling (*Pelargonium* × *hortorum* L.) susceptibility to *Pythium* infection or at least there is no enhancement in infection [32]. However, this may not represent what would occur under greenhouse conditions.

Fungus gnat adults are capable of carrying, on their bodies, the aerial conidia of certain foliar and soil-borne plant pathogenic fungi that produce aerial dispersal stages such as *Botrytis cinerea* Pers.:Fr., *F. avenaceum*, *F. acuminatum* (Ellis & Everhart), *T. basicola*, *V. dahliae* Kleb., and *V. albo-atrum*, which can then be transmitted to healthy plants [25,27,28,29,33]. Adult fungus gnats may not be able to vector *Pythium* spp., because any sexual and/or asexual reproductive structures are produced belowground (there are no aerial dispersal stages), which are not accessible to fungus gnat adults for acquisition because adults live aboveground [34,35].

Although adults do not fly very well and tend to reside near the growing medium, they may still be able to disperse spores of foliar plant pathogens in localized areas of the greenhouse [33]. The potential for both life stages of fungus gnats to transmit diseases means that the tolerance level for the presence of this pest may be very low, which is similar to other insect pests that vector diseases such as the western flower thrips, *Frankliniella occidentalis* Pergande [36]. Moreover, this means that intensive plant protection practices need to be implemented including alternative management strategies.

## 3. Alternative Management Strategies

Moisture management and sanitation are important in reducing problems with fungus gnats in greenhouse production systems. The use of well-drained growing media and not over-watering plants may avoid issues with soil-borne plant pathogenic fungi, thus diminishing the possibility of disease transmission by the larvae and adult. The accumulation of water and presence of algae may lead to abundant populations of fungus gnats and thus damage to greenhouse-grown crops [37]. Therefore, the installation of cement flooring may eliminate problems with algae, and consequently fungus gnats. However, this may not be economically feasible. A more cost-effective strategy may be to use landscape or weed fabric barriers, which are geotextile, non-biodegradable materials that when installed underneath benches may reduce algae growth and thus mitigate problems with fungus gnats [11]. It is also important to remove plant material and growing medium debris immediately from the greenhouse or place into containers, located within greenhouses, with tight-sealing lids, as fungus gnat adults may emerge from disposed growing medium and migrate onto the main crop [38].

An alternative strategy that should be considered in order to alleviate problems with fungus gnats is the use of physical barriers. Physical barriers are designed to alter the environment in order to make it inaccessible to insect pests [39]. Physical barriers do not reduce insect pest populations immediately, which is typical of most insecticides [40]; however, placing a physical barrier on the surface of the growing medium may inhibit fungus gnat adult emergence and also possibly reduce female egg-laying or egg survival [41].

It has been hypothesized that physical barriers such as sand or diatomaceous earth placed on the surface of the growing medium would negatively impact fungus gnat adults when they emerge and/or prevent females from laying eggs [42]. Diatomaceous earth is composed of siliceous skeletonized diatoms [43], which remove the insect cuticle waxes, absorb oils and waxes on the outer cuticle, or disrupt the integrity of the insect cuticle resulting in extensive loss of water from the insect body [44]. However, it has been shown that placing diatomaceous earth or sand on the growing medium surface did not have any effect on fungus gnat adult emergence or inhibit females from laying eggs because these physical barriers contain small openings that allow larvae to pupate, and adult females to lay eggs [41,45].

Growstones^™^ (Growstone, Inc., Albuquerque, NM, USA) aggregates, which are processed from 100% recycled glass and are used as a substrate in hydroponic systems [46] have been used as physical barriers and shown to reduce fungus gnat adult emergence over time and may also reduce egg-laying or egg survivability although this depends on the thickness of the Growstones^™^ layer [47]. It is important to note, that a physical barrier is not a stand-alone practice and therefore must be used as part of a holistic plant protection strategy [39]. This strategy may be more effective with plants growing in large containers whereas physical barriers may not be feasible under propagation due to the excessively moist conditions and small size of the containers.

Another potential alternative strategy to minimize plant damage due to fungus gnats is by repelling fungus gnat adults away from the growing medium thus reducing egg-laying. Repellent compounds have been used against other insects such as mosquitoes [48,49]. It was discovered, under laboratory conditions, that Bounce^®^ original brand fabric softener dryer sheets (Outdoor Fresh Scent; Procter and Gamble, Cincinnati, OH) actually repel fungus gnat adults [50]. The dryer sheets contain several volatile constituents including linalool (3,7-dimethyl-1,6-octadien-3-ol), a colorless monoterpene alcohol that is present naturally in plants such as lavender (*Lavandula angustifolia* Mill.), marjoram (*Origanum vulgare* L.), and basil (*Ocimum basilicum* L.) [51,52,53]. In fact, greenhouse producers are actually inserting dryer sheets into the growing medium to repel fungus gnat adults [11]. However, one problem with this strategy is that fungus gnat adults are not killed directly and may migrate into other areas of the greenhouse. Therefore, dryer sheets must be placed throughout the greenhouse in order to protect plants from fungus gnat adults.

Finally, the use of jasmonate has been investigated to determine if stimulating wound signaling pathways may protect plants against fungus gnat larvae. This is based on the premise that signaling pathways, when activated by insects with chewing mouthparts, may allow plants to defend themselves by producing secondary metabolites [54]. However, it was found that applications of methyl jasmonate to *Arabidopsis thaliana* plants resulted in no mortality or reduced feeding activity of fungus gnat larvae [55].

## 4. Conclusions

It is important to understand that fungus gnats, both larvae and adults, may transmit certain soil-borne plant pathogens (e.g., fungi) to greenhouse-grown horticultural crops, either directly or indirectly thus potentially resulting in an economic loss. Although insecticides and biological control agents are effective against fungus gnat larval populations, alternative plant protection strategies should also be implemented. This includes cultural, sanitation, physical barriers, and use of repellent materials. These strategies may contribute to enhancing the efficacy of insecticides and biological control agents in suppressing or regulating fungus gnat populations in greenhouse production systems.
